# Hollow Crystallization COF Capsuled MOF Hybrids Depict Serum Metabolic Profiling for Precise Early Diagnosis and Risk Stratification of Acute Coronary Syndrome

**DOI:** 10.1002/advs.202302109

**Published:** 2023-06-20

**Authors:** Chenjie Yang, Yilong Pan, Hailong Yu, Xufang Hu, Xiaodong Li, Chunhui Deng

**Affiliations:** ^1^ Department of Chemistry Fudan University Shanghai 200433 China; ^2^ Department of Cardiology Shengjing Hospital of China Medical University NO.36 Sanhao Street, Heping District Shenyang 110004 China; ^3^ School of Chemical Science and Technology Yunnan University No. 2 North Cuihu Road Kunming 650091 P. R. China

**Keywords:** acute coronary syndrome, acute myocardial infarction, covalent organic frameworks (COFs), metabolic profiling, metal–organic frameworks (MOFs)

## Abstract

Acute coronary syndrome (ACS), comprising unstable angina (UA) and acute myocardial infarction (AMI), is the leading cause of death worldwide. Currently, lacking effective strategies for classifying ACS hinders the prognosis improvement of ACS patients. Disclosing the nature of metabolic disorders holds the potential to reflect disease progress and high‐throughput mass spectrometry‐based metabolic analysis is a promising tool for large‐scale screening. Herein, a hollow crystallization COF capsuled MOF hybrids (UiO‐66@HCOF) assisted serum metabolic analysis is developed for the early diagnosis and risk stratification of ACS. UiO‐66@HCOF exhibits unrivaled chemical and structural stability as well as endowing satisfying desorption/ionization efficiency in the detection of metabolites. Paired with machine learning algorithms, early diagnosis of ACS is achieved with the area under the curve (AUC) value of 0.945 for validation sets. Besides, a comprehensive ACS risk stratification method is established, and the AUC value for the discrimination of ACS from healthy controls, and AMI from UA are 0.890, and 0.928. Moreover, the AUC value of the subtyping of AMI is 0.964. Finally, the potential biomarkers exhibit high sensitivity and specificity. This study makes metabolic molecular diagnosis a reality and provided new insight into the progress of ACS.

## Graphical abstract

A hollow crystallization COF capsuled MOF hybrids assisted LDI‐MS serum metabolic analysis platform is proposed. The high‐throughput serum metabolic profiling coupled with machine learning algorithms successfully achieves early diagnosis and risk stratification of acute coronary syndrome. Also, the subtyping of acute myocardial infarction is accomplished. This study provides a new insight into the high‐throughput metabolic molecular diagnosis.

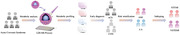

## 1 Introduction

Acute coronary syndrome (ACS), encompassing unstable angina (UA) and acute myocardial infarction (AMI), is defined as comprehensive clinical symptoms of severe myocardial ischemia and is regarded as the most lethal disease.^[^ ^1^ ^]^ UA is relatively less life‐threatening for patients induced by transitory myocardial ischemia; however, the majority of patients with UA will ultimately progress to AMI without early diagnosis and appropriate treatments.^[^ ^2^ ^]^ Further, AMI including ST‐segment elevation myocardial infarction (STEMI) and non‐ST‐segment elevation myocardial infarction (NSTEMI) is more fatal since it is accompanied by myocardial necrosis.^[^ ^3^ ^]^ The release of extensive myocardial necrosis markers such as cardiac troponin I (cTnI) and creatine kinase isoenzyme (CK‐MB) in AMI was caused by the arterial blockage devouring lives in minutes under delayed rescue conditions, and the most recent data estimated that more than 7 million new instances of AMI occurred globally in 2021.^[^ ^4^ ^]^ However, the diagnosis and risk stratification of the ACS at present are performed by the comprehensive assessment of issues involving patients' symptoms, eletrocardiogram (ECG), together with myocardial necrosis markers including cTnI, CK‐MB, etc., which is both time consuming and tiresome in emergency conditions.^[^ ^2^ ^]^ Besides, as the early symptom of ACS, UA is difficult to diagnose ascribed to the normal ECG results and no increase in cTnI and CK‐MB that can hardly be recognized even by experienced physicians.^[^ ^5^ ^]^ Moreover, the classification of UA and NSTEMI is another obstacle since patients with either of the disease present comparable physical symptoms and ECG results. Despite cTnI and CK‐MB have been widely used as the main biomarker to distinguish UA and AMI; however, more recently, studies have revealed that cTnI and CK‐MB disruption may be associated with other disorders, such as tachy‐ or brady‐arrhythmias and renal failure, which are not specific to AMI.^[^ ^6^ ^]^ At this point, developing a precise diagnosis and risk stratification of ACS is of great significance.^[^ ^7^ ^]^

Molecular diagnosis is a promising tool for clinical screening that avoids misdiagnosis due to the physicians' empirical judgments and provides a rational interpretation of disease progression from a molecular perspective.^[^ ^8^ ^]^ Metabolites, as the end products of life activities including genetic information and protein macromolecule expression, reflect the most natural state of the organism, thus great efforts have been devoted to the discovery of biomarkers for early diagnosis.^[^ ^9^ ^]^ Mass spectrometry featuring high sensitivity, high throughput, and molecular identification ability with the matched database has developed into a powerful tool for metabolic analysis.^[^ ^10^ ^]^ In particular, liquid chromatography–mass spectrometry (LC–MS),^[^ ^11^ ^]^ and gas chromatography–mass spectrometry (GC–MS)^[^ ^12^ ^]^ have achieved huge success in disease diagnosis, especially for disease‐related metabolic pathway analysis and biomarkers discovery.^[^ ^13^ ^]^ However, for large‐scale screening, the application of these methods is limited by their time‐consuming analysis, and developing ultra‐high throughput mass spectrometry techniques is in urgent need. Matrix‐assisted laser desorption/ionization–mass spectrometry (MALDI–MS) seems to perfectly meet such disease screening requirements due to its advantages of nanoscale sample loading, seconds‐like analysis speed, and the high throughput result readout.^[^ ^14^ ^]^ Nevertheless, although MALDI–MS has significantly promoted the advancement in identifying large molecules such as proteins and peptides,^[^ ^15^ ^]^ its applications in small molecular detection profiling are hampered by the overlapping low‐molecular region produced by the traditional organic substrates.^[^ ^16^ ^]^ In the past decades, inorganic substrates such as carbon nanomaterials,^[^ ^17^ ^]^ titanium oxide^[^ ^18^ ^]^ and noble metal^[^ ^19^ ^]^ have inspired the detection of small molecules by MALDI–MS. The stable chemical structure and efficient ionization efficiency promise the detection of the key metabolites which are generally at ultralow concentration. Metal–organic frameworks (MOFs) and covalent organic frameworks (COFs), exhibiting large surface area, high crystallinity, and controlled nanopores structures, have attracted considerable attention in photocatalysis,^[^ ^20^ ^]^ molecular sensing,^[^ ^21^ ^]^ gas storage,^[^ ^22^ ^]^ and diagnosis of diseases.^[^ ^23^ ^]^ Notably, MOFs have been considered promising substrates for numerous affinity sites for the metabolites, and the metal ions in MOFs have been elucidated to be beneficial to charge transfer during the ionization process.^[^ ^24^ ^]^ Comparatively, the covalent bonds between organic monomers endow COFs with enhanced chemical stability than MOFs.^[^ ^25^ ^]^ Besides, the extended *π*–*π* structures in COFs are conducive to improving ionization efficiency as the substrate of MALDI–MS.^[^ ^23^, ^26^ ^]^ More recently, composites with an advanced structure like core‐shell or yolk‐shell structures enjoy enhanced ion efficiency, and the integration of MOFs and COFs holds impressive forward.^[^ ^27^ ^]^ However, the traditional harsh synthesis conditions of COFs^[^ ^28^ ^]^ and the grand challenges in the design of yolk–shell structure‐like structure templates limit the application of yolk–shell MOF@COF.^[^ ^29^ ^]^

**Scheme 1:**
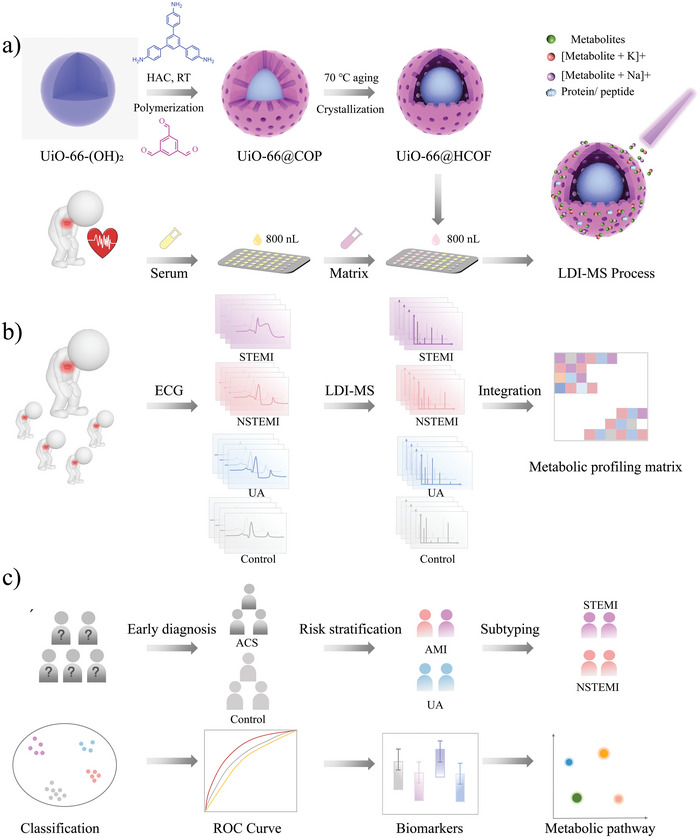
The workflow of UiO‐66@HCOF assisted LDI‐MS metabolic analysis system.

Herein, a novel hollow crystallization COF capsuled MOF hybrid (UiO‐66@HCOF) was proposed as an alternative substrate of LDI‐MS. Then, we developed a UiO‐66@HCOF‐assisted LDI‐MS metabolic profiling analysis system for precise early diagnosis and risk stratification of ACS (**Scheme** **1**). As displayed in Scheme 1a, UiO‐66‐(OH)_2_ was adopted as the core and initially assembled with a covalent organic polymer coating layer. After facile aging treatment, a hollow COF capsuled UiO‐66‐(OH)_2_ was constructed and donated as UiO‐66@HCOF. Compared to the traditional core–shell structure, a yolk–shell structure like UiO‐66@HCOF retains the intrinsic properties of the MOFs and COFs to the maximum degree and fully exposes the large active sites of both. Only 800 nL of serum are required, and the metabolic profiling is obtained as Scheme 1b displayed. In this work, UiO‐66@HCOF successfully extracted 211 metabolic profiling, including 41 patients with NSTEMI, 57 patients with STEMI, 70 patients with UA, and 53 healthy controls (CON). As shown in Scheme 1c, combined with machine learning algorithms, this system accomplished the early diagnosis of ACS with the area under the curve (AUC) value of 0.945 for the validation test. Besides, this work achieved the risk stratification of ACS and classify in seconds from ACS to AMI and UA as well as from AMI to NST and ST. Significantly, key metabolites were screened out and employed as biomarkers for diagnosing different types of ACS. In this work, the UiO‐66@HCOF assisted LDI‐MS metabolic profiling analysis system features both ultra‐fast analysis speed for early diagnosis and risk stratification of ACS and provides a relatively non‐invasive detection strategy for personalized treatment of patients with ACS.

## 2 Results and Discussion

### 2.1 The Synthesis and Characterization of UiO‐66@HCOF

**Figure 1:**
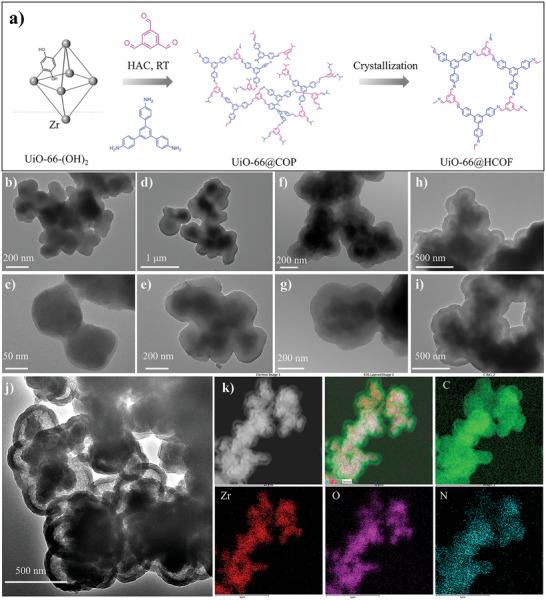
The design and morphological characterization of UiO‐66@HCOF. The synthesis strategy of UiO‐66@HCOF (a). TEM images of UiO‐66‐(OH)_2_ (b, c), UiO‐66@COP (d, e). TEM image of the UiO‐66@COP after aging 24h (f, g). TEM image of UiO‐66@COP after aging for 48 as well as UiO‐66@HCOF (h, i). The FETEM of UiO‐66@HCOF (j). The element mapping of UiO‐66@HCOF and the related elemental distribution (k).

The synthesis of UiO‐66@HCOF was constructed through a strategy of aging after assembly under mild conditions, which consists of the following three simplified steps (**Figure** **1**a). Initially, the UiO‐66‐(OH)_2_ was synthesized according to previous work with slight modification and employed as the core component.^[^ ^30^ ^]^ Following this, 1,3,5‐benzenetricarboxaldehyde (BTCA) and 1,3,5‐tris(4‐aminophenyl)benzene (TAPB) assembled to covalent organic polymer (COP) coating on UiO‐66‐(OH)_2_ to fabricate UiO‐66@COP. Eventually, UiO‐66@COP was aged at a higher temperature to generate UiO‐66@HCOF hybrids.^[^ ^31^ ^]^ The morphology of the three products is studied by transmission electron microscope (TEM). As Figure 1b,c displays, UiO‐66‐(OH)_2_ presented a uniform distribution with a diameter of about 120 nm which is consistent with the previously reported literature.^[^ ^30^ ^]^ Then, a COP coating layer around 30 nm thick can be observed on the surface of UiO‐66‐(OH)_2_ from Figure 1d,e, demonstrating the successful assembling of UiO‐66@COP. We further investigated the hollow crystalline structure transformation process from UiO‐66@COP to UiO‐66@HCOF after aging treatment. As shown in Figure 1f,g, the outer COP layer becomes dense and compact after aging for 24 h, indicating the occurrence of the structural transformation from amorphous to crystalline. After the 48 h aging, the ultimate UiO‐66@HCOF products, exhibiting yolk‐shell structure companies with hollow crystalline structure were completely formed (Figure 1h,i) which is consistent with the TEM characterization of COP to HCOF in Figure S1 (Supporting Information). Notably, from the field emission transmission electron microscopy (FETEM), UiO‐66@HCOF preserved the complete original structure of UiO‐66‐(OH)_2_, and the crystallization COF layer as thin hollow spheres wrapped around the UiO‐66‐(OH)_2_ (Figure 1j). These results demonstrated the disordered and cross‐linked frameworks of COPs had been transformed to crystalline imine networks to obtain a superior stable nanostructure. Moreover, the element mapping is also characterized and shown in Figure 1k, the Zr and the O elements that mainly belong to the UiO‐66‐(OH)_2_ clearly occupied the core structure, and the C and N elements are mostly distributed in the hollow shell structure. Thus, the exquisite structure and well‐maintenance of the chemical composition of UiO‐66@HCOF provide a powerful guarantee for the synergistic ionization effect of MOF and COF in subsequent LDI‐MS analysis.

**Figure 2:**
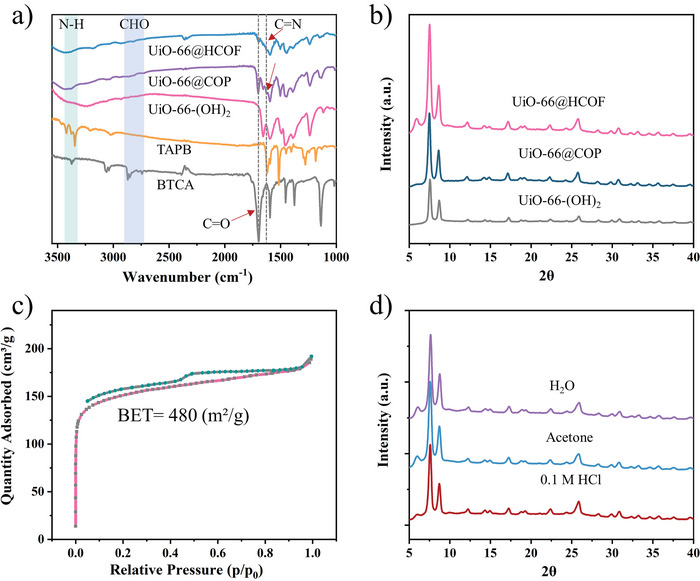
The characterization of UiO‐66@HCOF. a) The FT‐IR spectra of BTCA, TAPB, UiO‐66‐(OH)_2_, UiO‐66@COP, and UiO‐66@HCOF. b) The PXRD patterns of UiO‐66‐(OH)_2_, UiO‐66@COP, and UiO‐66@HCOF. c) The N_2_‐adsorption and desorption isotherm of UiO‐66‐@HCOF. d)The PXRD patterns of UiO‐66@HCOF after being treated with H_2_O, acetone, and 0.1 m HCl for 24 h.

The Fourier transform‐infrared spectra (FT‐IR) were tested to demonstrate the changes in the composition of the material products at each synthesis step. As displayed in **Figure** **2**a, with the formation of the COP and COF layer, the stretching vibrations (3343 and 3420 cm^−1^) of —N—H of TAPB disappeared, and the —C=O (1700 cm^−1^) of BTCA can still be observed in UiO‐66@COP and UiO‐66@HCOF that probably belongs to the remaining aldehyde end‐group of COP or COF. Notably, the signal of —C=N—(1620 cm^−1^) generated between the —C=O carboxylic acid functional group (1650 cm^−1^) of UiO‐66‐(OH)_2_ and the —C=C (1590 cm^−1^) of the stretching vibration from benzene skeleton. These results verifying most of the —N—H and —C=O groups are successfully reacted to form an ordered imine chemical bond structure. The crystalline structures of the prepared materials were studied using powder X‐ray powder diffraction (PXRD). As illustrated in Figure 2b, the results indicate the successful preparation of UiO‐66‐(OH)_2,_ and the coated COP layer maintained the high crystallization UiO‐66‐(OH)_2_. However, different from UiO‐66@COP, UiO‐66@HCOF exhibited representative diffraction peaks of HCOF at 5.7° that demonstrated the formation of crystallization after the aging. The PXRD patterns of UiO‐66@HCOF aging for 12, 24, 36, and 48 h were also recorded, and as Figure S2 (Supporting Information) displayed, UiO‐66@HCOP featured 5.7° diffraction peaks at 12 h that illustrated the formation process of crystalline structure. Notably, during the complete aging process, UiO‐66‐(OH)_2_ maintained the original diffraction peaks without being affected.

Besides, the specific surface area was estimated by N_2_‐adsorption and desorption isotherms, as shown in Figure S3a (Supporting Information), UiO‐66‐(OH)_2_ showed type I isotherm and Brunauer–Emmett–Teller (BET) surface area is 436 m^2^ g^−1^. As Figure 2c displays, UiO‐66@HCOF also exhibited typical type I isotherm, and BET surface area is 480 m^2^ g^−1^ that illustrated the enhancement of specific surface area with the formation of the HCOF layer. Moreover, in Figure S3b (Supporting Information), UiO‐66@HCOF preserved the pore properties of UiO‐66‐(OH)_2_ that indicates UiO‐66‐(OH)_2_ is not being affected during the aging treatments. As Figure 2d displays, the chemical and structural stabilities of UiO‐66@HCOF were tested after being treated with various solvent environments for 24 h and the results illustrated that UiO‐66@HCOF exhibited perfect performance in aqueous solutions, organic solvents, and acidic solutions.

### 2.2 The Mechanism Study and Typical Metabolites Detection by LDI–MS Metabolic Analysis System

The mechanism study of nanomaterials in the desorption/ionization (D/I) process is significant for component selection as well as advanced structural design.^[^ ^32^ ^]^ Based on the reported literature, three major factors including UV–vis light absorption, thermal effects, and charge transfer have been studied in the D/I process to investigate the mechanism in the LDI–MS process. First, the UV–vis light absorption capability of UiO‐66@HCOF, HCOF, and UiO‐66‐(OH)_2_ at 355nm (the wavelength of the MALDI‐equipped laser) was estimated. As shown in Figure S4 (Supporting Information), UiO‐66@HCOF has a higher absorption at 355nm than UiO‐66‐(OH)_2_ and HCOF, indicating that UiO‐66@HCOF can utilize enhanced UV laser light energy from substrate to analytes in the D/I process.^[^ ^32^ ^]^ The thermal effects in the D/I process were then investigated, and the chemical thermometer benzyl pyridinium ion (BP^+^) was used as a probe to estimate the extent of thermal transfer from substrate to analytes.^[^ ^33^ ^]^ Under thermal effects, BP^+^ can dissociate into a benzyl cation [BP‐ Pyridine]^+^ and pyridine, as shown in Figure S5a (Supporting Information). The total BP^+^ intensity, which includes the intensity of [BP]^+^ and [BP‐ Pyridine]^+^, summarizes thermal and non‐thermal effects of D/I. Survival yield (SY) is defined as BP^+^/(BP^+^+[BP‐ Pyridine]^+^) and has an inverse relationship with thermal effects absorption. The mass spectra of BP^+^ using UiO‐66@HCOF, UiO‐66‐(OH)_2_, and HCOF are displayed in Figure S5b (Supporting Information) and the total BP^+^ intensity and SY are also displayed in Figure S5c (Supporting Information). The total BP^+^ intensity follows UiO‐66@HCOF> HCOF >UiO‐66‐(OH)_2_. However, the SY presents UiO‐66@HCOF< HCOF < UiO‐66‐(OH)_2_. The results show that UiO‐66@HCOF has the best thermal effects D/I, and HCOF also has satisfying thermal effects of D/I. Charge transfer is another key factor in the D/I process that cannot be ignored.^[^ ^34^ ^]^ Nanomaterials excited by UV lasers can generate electron–hole pairs, and the separated electron–hole in nanomaterials is expected to facilitate ionization in analytes through enhanced charge transfer.^[^ ^35^ ^]^ However, the visualization and evaluation of electron–hole separation are always of great interest to researchers. We performed the photocurrent‐time curves of UiO‐66@HCOF, HCOF, and UiO‐66‐(OH)_2_ to character the capability of electron‐hole separation (EHS). Figure S6 (Supporting Information) shows that UiO‐66@HCOF has higher EHS than HCOF and UiO‐66‐(OH)_2_. More intriguingly, the HCOF exhibits low EHS compared to UiO‐66‐(OH)_2_, which may be due to the absence of metal nodes in the structures.^[^ ^24a^ ^]^ To further illustrate the EHS capability, we used tetrabutylammonium cation (TBA^+^) as a sensitive probe to the generation of electrons and holes.^[^ ^36^ ^]^ As shown in Figure S7 (Supporting Information), the UiO‐66@HCOF displayed the highest TBA^+^ intensity also demonstrated the efficient charge transfer in the D/I process. Besides, the TBA^+^ intensity of UiO‐66‐(OH)_2_ is higher than that of HCOF which is consistent with the photocurrent‐time results. Based on the above results, both HCOF and UiO‐66‐(OH)_2_ enjoyed favorable UV–vis light absorption. HCOF characterizes a satisfying thermal effect,^[^ ^37^ ^]^ and UiO‐66‐(OH)_2_ is more efficient in charge transfer.^[^ ^38^ ^]^ As a result, UiO‐66@HCOF combines the benefits of UiO‐66‐(OH)_2_, and HCOF. The D/I process of UiO‐66@HCOF is co‐promoted with the comprehensive interactions of UV–vis light absorption, thermal effects, and charge transfer.

**Figure 3:**
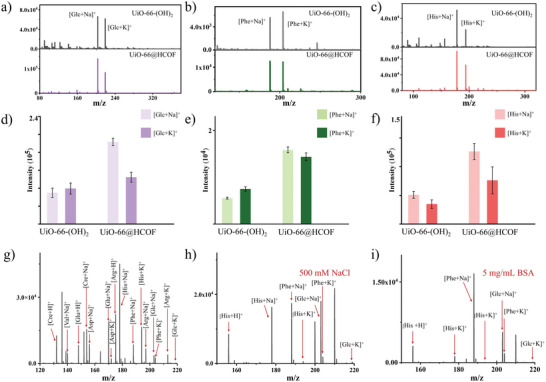
Detection of the typical metabolites by LDI‐MS system with UiO‐66‐(OH)_2_ and UiO‐66@HCOF as substrates. The detection of a) Glc (1 mg mL^−1^), b) Phe (0.02 mg mL^−1^), and c) His (1 mg mL^−1^). The corresponding adducts [Glc+Na]^+^ and [Glc+K] ^+^ (d), [Phe+Na]^+^ and [Phe+K] ^+^ (e), and [His+Na]^+^ and [His+K]^+^ (f) (*n* = 8). The mixture of the eight typical metabolites including the Cre, Val, Glu, Asp, His, Phe, Arg, and Glc with an average concentration is 0.1 mg mL^−1^ (g). Detection of the mixture of the Asp, Phe, and Glc in 500 mm NaCl solution (h). Detection of the mixture of the Asp, Phe, and Glc in 5 mg mL^−1^ bovine serum albumin solution (i).

As the advanced hybrids of MOFs and COFs as well as the highly crystalline COP, the performance of UiO‐66@HCOF as LDI–MS substrate for metabolite analysis was comprehensively investigated. First, to ensure less background interference generated by self‐desorption of the substrate under laser irradiation, the background interference was estimated. The results of direct LDI‐MS detection (Figure S8, Supporting Information) of UiO‐66‐(OH)_2_, UiO‐66@HCOF, and traditionally organic matrix of 2,5‐dihydroxybenzoic acid (DHB) evidenced that both UiO‐66‐(OH)_2_ and UiO‐66@HCOF have a clean background. Afterward, the detection of typical metabolites as promising serum biomarkers like glucose (Glc), l‐phenylalanine (Phe), and histidine (His) were performed to determine the performance of the substrates. The mass spectra of these metabolites (**Figure** **3**a–c) and their matched average intensities of the corresponding Na^+^ and K^+^ adducts (Figure 3d–f) showed that UiO‐66@HCOF produces enhanced metabolites signals as substrate compared to UiO‐66‐(OH)_2_. UiO‐66‐(OH)_2_ exhibited some matrix noise around the 100 m/z region which is probably caused by the non‐covalent binding between metal ions and organic ligands. In Figure S9, the detection performance of HCOF was also estimated. Compared to UiO‐66‐(OH)_2_, HCOF showed nearly ionization ability to UiO‐66‐(OH)_2_, and UiO‐66@HCOF still shows superior detection performance to HCOF. Hence, on the one hand, the HCOF layer coated on the UiO‐66‐(OH)_2_ imparts higher structure stability to the hybrid, which maintains thermal and chemical stability during the LDI‐MS process. On the other hand, UiO‐66@HCOF endows excellent D/I ability that is consistent with the UV–vis light absorption, thermal effects, and charge transfer evidence.

We further examined the D/I ability of UiO‐66@HCOF by expanding the number of analytes. As shown in Figure 3g, a mixture containing eight typical metabolites including creatine (Cre), valine (Val), glutamic acid (Glu), aspartic acid (Asp), His, Phe, arginine (Arg), and Glc, with an average concentration of 0.1 mg mL^−1^, is detected by the LDI–MS system. As expected, all the metabolites were detected with an impressive intensity of their corresponding adducts. Moreover, high salt concentration in the actual serum sample can cause serious interference in the detection of metabolites, so the salt tolerance was evaluated. As displayed in Figure 3h, the mixed metabolites of the Glc, Phen, and Asp (with an average concentration of 0.25 mg mL^−1^) were successfully detected in 500 mm NaCl solution. Likewise, the abundant biomolecules such as proteins and peptides pose a grand challenge to the detection of metabolites. In this work, despite concentrations of bovine serum albumin (BSA) up to 5 mg mL^−1^, the clear *m*/*z* features, and the excellent signal‐to‐noise ratios of adducts belonging to Glc, Phe, and Asp are obtained (Figure 3i). Further, to test the reproducibility of the metabolite analysis system, 40 times of LDI–MS results were recorded for Phe, Arg, and Asp and the corresponding RSD values are 12.3%, 17.5%, and 13.6% (Figure S10, Supporting Information). The signal intensities of these three metabolites are essentially at stable levels with a very small fluctuation, demonstrating the reliability and high reproducibility of our system.

### 2.3 Serum Metabolic Profiling for Early Diagnosis and Risk Stratification of Acute Coronary Syndrome

**Figure 4:**
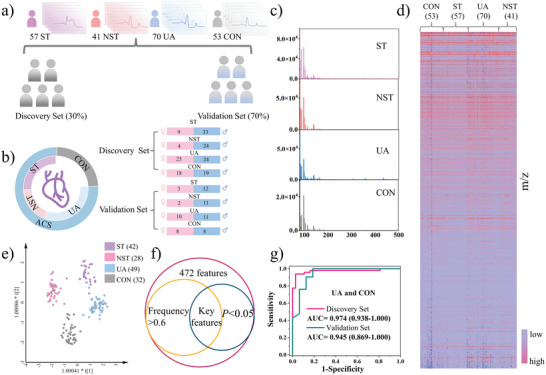
Clinical information for the patients and early diagnosis of ACS. a) Number of trialists from ST, NST, UA, and CON groups as well as the division of train set, and validation set. b) the classification of groups and the gender distribution in ST, NST, UA, and CON group. c) The typical serum metabolic profiling of ST, NST, UA, and CON depicted by the LDI–MS system. d) Matrix of 221 metabolic profiling including 53 CON, 57 ST, 70 UA, and 41 NST. e) OPLS‐DA for the discrimination of ST, NST, UA, and CON. f) The filtering rules of key features in various groups. g) ROC curve of the discovery set and validation set of ACS and CON.

Extracting high‐quality metabolic fingerprints is an essential step in subsequent metabolic diagnosis as well as metabolic biomarker discovery. We validated the satisfying metabolic profiling detection ability of the established UiO‐66@HCOF‐assisted LDI‐MS system in practical bio‐samples by employing an ACS patient serum and a CON individual serum. Also, the same analysis was carried out with UiO‐66‐(OH)_2_ and HCOF as substrates for comparison. As expected, whether measured on the ACS patient or the CON individual, UiO‐66@HCOF both exhibited richer metabolic profiling and enhanced metabolite signals (Figure S11, Supporting Information). Based on this system, we attempt to screen out serum metabolic biomarkers for the early diagnosis and the risk stratification of ACS. Clinical serum samples from 221 trial participants were collected in this study, including 57 patients with STEMI (ST), 41 patients with (NST), 70 patients with UA, and 53 healthy participants as controls (CON). As shown in **Figure** **4**a–b, these 211 participants were randomly divided into the discovery and validation sets, accounting for ≈70% and 30% of each group, respectively. The analysis of variance (ANOVA) and chi‐square tests were performed to evaluate the age and gender differences between the three groups, and the results were listed in Table S1 (Supporting Information). Statistical analysis suggested that there is no significant difference in age (*p* > 0. 05) in the three groups. However, there exists a significant difference in gender between AMI (*p <* 0.05) and UA/CON indicating that AMI more probably occurs in males that is consistent with the reported literature.^[^ ^39^ ^]^ Therefore, in this work, the gender difference in the AMI sample collection is relatively reasonable. Following this, 800 nL of diluted serum were analyzed by the UiO‐66@HCOF‐assisted LDI‐MS metabolic analysis system, and the presentative metabolic profiling spectra are shown in Figure 4c. The original mass spectra were extracted and processed with peak alignment, average, and normalization and each metabolic profiling include 472 features. As Figure 4d displays, an overall metabolic profiling matrix is formed, and it can be preliminarily seen that four groups produced specific metabolic features. Orthogonal partial least squares discriminant analysis (OPLS‐DA) is the most intuitive statistical tool and is widely applied for the classification of metabolic profiling matrix with one or more classes.^[^ ^40^ ^]^ We initially applied OPLS‐DA to preliminary characterize the metabolic difference between the four groups. The results yielded by the OPLS‐DA model (*R* ^2^ *Y* = 0.891, *Q* ^2^ = 0.617) in Figure 4e exhibited that UA, ST, NST, and CON occupied four different parts in the 95% confidence interval and diverged in the exact opposite direction. Moreover, the 200 permutation results of this model were displayed in Figure S12 (Supporting Information) where the intersection points between both lines and the *y*‐axis are lower than the upper right value, illustrating the high reliability of this model. It further demonstrated that though the patients with AMI or UA suffered similar symptoms, metabolic profiling can tap into the changes of different ACS subtypes at the molecular level and provide the chance to discover the biomarkers. Random forest (RF) algorithm has been proven and widely employed as a credible machine‐learning approach for screening biomarkers in massive metabolic features.^[^ ^41^ ^]^ This work employed RF algorithm for the screening of the key features between the various groups according to the displayed criteria in Figure 4f that the frequency value is more than 0.6, together with the *p*‐value less than 0.05. The key features between different groups were displayed in Tables S2–S7 (Supporting Information) and the subsequent machine‐learning analysis is based on these key features.

**Figure 5:**
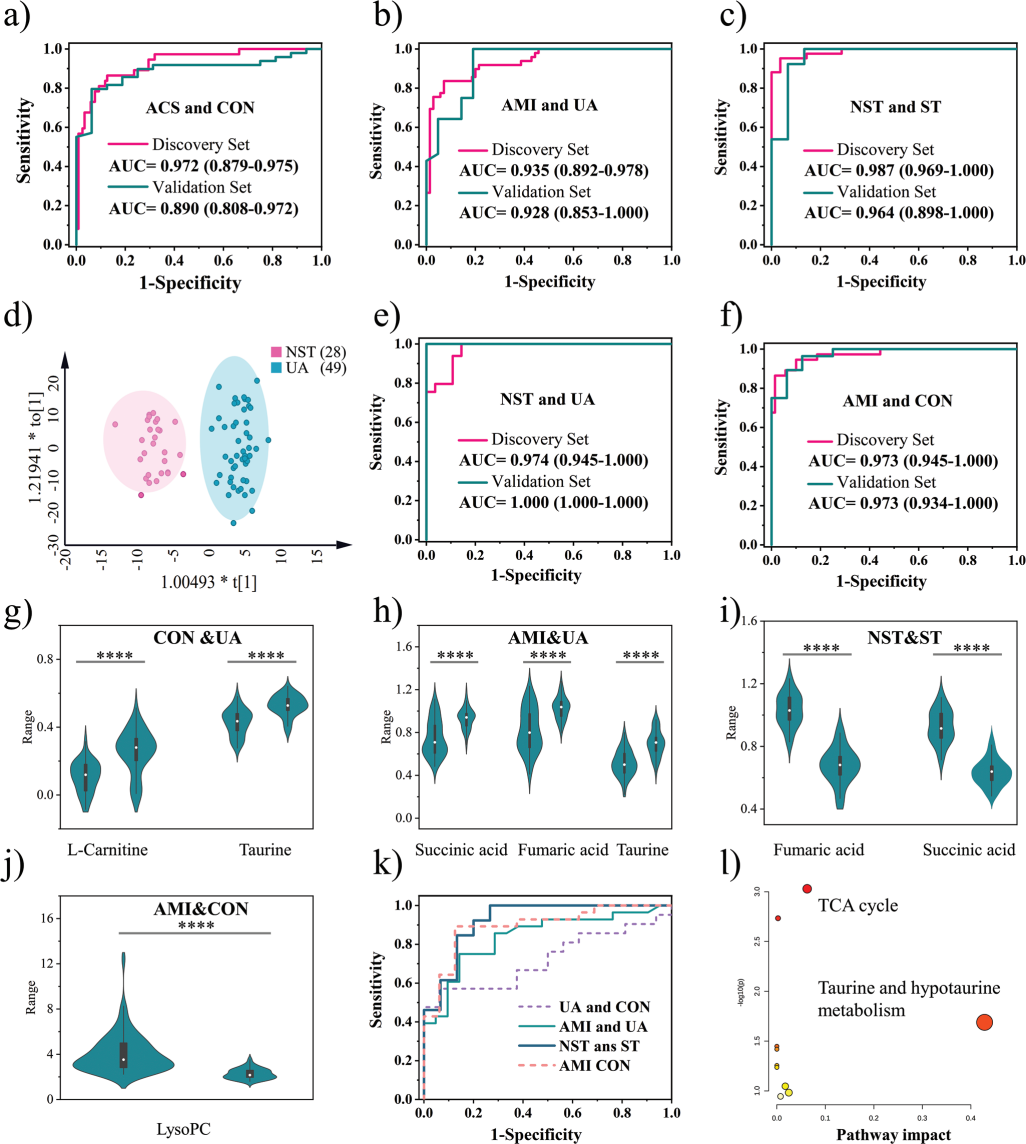
Risk stratification of ACS and the screening of biomarkers. a) The ROC curve of initial diagnosis of ACS from CON. b) The ROC curve of risk stratification for AMI and UA. c) The ROC curve of subtyping AMI to NST and ST. d) OPLS‐DA model for the discrimination of NST and UA. e) The ROC curve of NST and UA. f) The ROC curve of AMI and CON. g) l‐arnitine, and taurine as the biomarkers for CON and UA. h) Succinic acid, fumaric acid and taurine as the biomarkers for AMI and UA. i) Succinic acid and fumaric acid as the biomarkers for NST and ST. j) LysoPC as the biomarkers for AMI and CON. The significant difference between the two groups is presented by ^****^ *p* < 0.001. k) The ROC curves of the validation set of UA&CON, AMI&UA, NST&ST, and AMI&CON with the screened‐out biomarkers. l) Metabolic pathways for the occurrence of ACS.

As already mentioned, UA is the early type of ACS that is difficult to diagnose by conventional clinical diagnostic methods. However, in this work, 8 screened‐out key features were employed to discriminate UA from CON and the receiver operating characteristic curve (ROC) is displayed in Figure 4g, of which the AUC value of 0.974 (95% CI: 0.938–1.000) for discovery set and 0.945 (95% CI: 0.928‐0.996) for the validation set which illustrated the powerful feasibility of this method in the early diagnosis of ACS. In addition to early diagnosis of ACS, risk stratification of ACS is critical for the precise and personalized treatment of patients. In this work, a complete diagnosis system was established. Firstly, the ROC curves were generated for the diagnosis of the ACS from CON. As displayed in **Figure** **5**a, a satisfying AUC value of 0.972 (95% CI: 0.879–0.975) with 86.5% sensitivity and 87.4% specificity for the discovery set. Likewise, comparable prediction accuracy was obtained for the validation set, of which the AUC is 0.890 (95% CI: 0.808–0.972) with 79.6% sensitivity and 93.8% specificity. Considering the great probability of UA progressing to life‐threatening AMI, the risk stratification between them is essential for precise diagnosis and guided treatment. As depicted in Figure 5b, the discrimination was achieved with the AUC value of the discovery set being 0.935 (95% CI: 0.892‐0.978) with 83.7% sensitivity and 92.9% specificity, and that of the validation set also reached 0.928 (95% CI: 0.853–1.000) with 100% sensitivity and 82% specificity. Besides, the precise recognition of each subtype of AMI by metabolic profiling is also anticipated that cannot only directly influence the determination of treatment protocols, but also can provide an insight into the progress of ST and NST from a metabolic molecular perspective. In Figure 5c, the AUC value of the discovery set is 0.987 (95% CI: 0.969‐1.000) with 95.2% sensitivity and 96.4% specificity, and that of the validation set is 0.964 (95% CI: 0.898–1.000) with 100% sensitivity and 86.7% specificity. In clinical practice, the depressing nature of most NST segments on the ECG shows similar findings to angina pectoris in patients with UA, thus often leading to high mortality in patients with NST because of the inability to distinguish between UA and NST patients in an emergency condition. To resolve this issue, we explored the metabolic differences between NST and UA patients. As Figure 5d displayed, the OPLS‐DA (*R* ^2^ *Y* = 0.918, *Q* ^2^ = 0.803) classification and the permutations in Figure S13 (Supporting Information) illustrated the significant difference between NST and UA. Besides, the ROC curves also showed an excellent diagnosis performance. As shown in Figure 5e, the AUC value of the discovery set between UA and NST is 0.974 (95% CI: 0.945–1.000) with 100% sensitivity and 85.7% specificity, and that of the validation set is 1 (95% CI: 1.000–1.000) with 100% sensitivity and 100% specificity. Finally, in Figure 5f, the AUC value of the discovery set and the validation set between AMI and CON are both 0.973. These results validated the robust typing identification ability of our metabolic profiling.

Based on the above analysis, the screened key features were identified according to the human metabolome database (HMDB), and the promising biomarkers were assigned to metabolites in Table S8 (Supporting Information). In Figure 5g, the l‐carnitine (*m*/*z* 162.11, HMDB0000062) and taurine (*m*/*z* 147.926, HMDB0000251) presented up‐regulated in UA compared with CON that is consistent with previous studies that higher l‐carnitine has recognized to be relevant to cardiovascular disease^[^ ^42^ ^]^ and widely discovered as the biomarkers for the diagnosis of AMI and the risk stratification of ACS.^[^ ^43^ ^]^ Moreover, studies have suggested that taurine may have neuro‐ and cardio‐protective functions, and the upregulated taurine in UA may be relevant to the neurological outcomes.^[^ ^44^ ^]^ Additionally, fumaric acid (*m*/*z* 154.881, HMDB0000134), succinic acid (*m*/*z* 156.897, HMDB0000254), and taurine (*m*/*z* 147.926, HMDB0000251) were detected down‐regulated in AMI in comparison to UA in Figure 5h.

More interestingly, as displayed in Figure 5i, in the subtyping of AMI, fumaric acid and succinic acid present upregulated in NST. Moreover, the lysophospholipid of LysoPC (22:5) (*m*/*z* 592.312, HMDB0010403) was considered to be upregulated in patients (Figure 5j). Afterward, the above potential biomarkers were employed for the diagnosis of the relevant types of ACS in Figure 5k. For instance, l‐carnitine and taurine between UA and CON achieved AUC values of 0.723 (95% CI: 0.558–0.888) for the discovery cohort. Likewise, the AUC values for the discovery cohort of AMI and UA, NST, and ST, and AMI and CON are 0.840 (95% CI: 0.729–0.953), 0.923 (95% CI:0.826–1.000), and 0.895 (95% CI:0.728–0.953), respectively. These results illustrated the high accuracy and specificity of this method for the early diagnosis and risk stratification of ACS. Finally, these potential biomarkers were integrated to explore metabolic pathway disorders in the occurrence of ACS. As displayed in Figure 5l, the metabolic disorders of the citrate cycle (TCA cycle) and taurine and hypotaurine metabolism are the main causes of ACS.

## 3 Conclusion 

In conclusion, this work developed a UiO‐66@HCOF‐assisted LDI‐MS metabolic analysis system for precise diagnosis and risk stratification of ACS. The UiO‐66@HCOF combined the advantages of UiO‐66‐(OH)_2_ and the hollow crystallization COF layer whose chemical structure brings robust stability and high ionization ability to the hybrids. Benefitting from the synergy effect of UiO‐66(OH)_2_ and hollow crystallization COF layer, metabolic profiling spectra were successfully extracted from 221 serum samples assigned to ST, NST, UA, and CON groups. Remarkably, OPLS‐DA intuitively classified the various groups, and the key metabolite features were screened through random forests machine learning algorithms. Notably, the early diagnosis of ACS was achieved from the discrimination between UA and CON, with the AUC value of the validation sets being 0.945. Besides, a complete ACS risk stratification system was established, and the discrimination of ACS and CON, and AMI versus UA were accomplished with satisfying AUC values of 0.890, and 0.928 for validation sets, respectively. Similarly, for precise AMI subtype stratification, the corresponding AUC values of validation sets demonstrated excellent prediction capability that the AUC value for NST versus ST is 0.985 with 100% sensitivity and 86.7% specificity. Moreover, the AUC value for NST and UA is 1 with 100% sensitivity and 100% specificity. Additionally, referred to the HMDB, the key features are assigned to the metabolites which were employed as the biomarker for the diagnosis of types of ACS. Finally, the potential biomarkers anticipant the significant metabolic pathways, and the metabolic disorders in the TCA cycle are regarded as the main cause of ACS. This work reveals the great promising advanced nanostructure‐based metabolic profiling analysis in disease diagnosis and stratification and demonstrates the great significance of metabolic analysis in the molecular subtyping of diseases.

## Conflict of Interest

The authors declare no conflict of interest.

## Supporting information

Supporting Information

## Data Availability

The data that support the findings of this study are available from the corresponding author upon reasonable request.
