# Supplementary material for: Hollow Crystallization COF Capsuled MOF Hybrids Depict Serum Metabolic Profiling for Precise Early Diagnosis and Risk Stratification of Acute Coronary Syndrome
Source: Adv Sci (Weinh). 2023 Jun 20;10(24):2302109. doi: 10.1002/advs.202302109 (PMC10460873; doi:10.1002/advs.202302109)
Supplement: Supplementary file 1 — Supporting Information [file ADVS-10-2302109-s001.pdf]

ADVANCED SCIENCE
Open Access

Supporting Information

for *Adv. Sci.*, DOI 10.1002/advs.202302109

Hollow Crystallization COF Capsuled MOF Hybrids Depict Serum Metabolic Profiling for Precise Early Diagnosis and Risk Stratification of Acute Coronary Syndrome

Chenjie Yang, Yilong Pan, Hailong Yu, Xufang Hu, Xiaodong Li* and Chunhui Deng**

Supporting Information

Hollow Crystallization COF Capsuled MOF Hybrids Depict Serum Metabolic Profiling for Precise Early Diagnosis and Risk Stratification of Acute Coronary Syndrome

Chenjie Yang,[+] Yilong Pan,[+] Hailong Yu, Xu fang Hu, Xiaodong Li*, Chunhui Deng**

C. Yang, Dr. H. Yu, Prof. C. Deng

Department of Chemistry, Fudan University, Shanghai, 200433, China.

E-mail: chdeng@fudan.edu.cn

Y. Pan, Prof. X. Li

Email: lixd@sj-hospital.org

Department of Cardiology, Shengjing Hospital of China Medical University, NO.36 Sanhao Street, Heping District, Shenyang, China.

Dr. X. Hu

School of Chemical Science and Technology, Yunnan University, No. 2 North Cuihu Road, Kunming, P. R. China, 650091，

Email: huxufang@ynu.edu.cn

Keywords: MOFs, COFs, acute coronary syndrome, acute myocardial infarction, metabolic profiling

Experimental Section/Methods

Chemicals and Reagents: All the chemicals and reagents are purchased at analytical grade without any purification during the experimental process. 1,3,5-Tris(4-aminophenyl) benzene (TAPB) and 1,3,5-benzenetricarboxaldehyde (BCTA) were purchased from the Yanshen Technology Co., Ltd (Jilin, China). L-Phenylalanine, L-Valine, L-Aspartic acid, creatine monohydrate, L-Arginine, D-Glutamic acid, L-Histidine, and glucose were purchased from Sigma-Aldrich. 2,5-dihydroxybenzoic acid (DHB) was purchased from Adamas.

The synthesis of UiO-66(OH)$_2$: The synthesis of UiO-66(OH)$_2$ is according to the reported method with slight modifications.[1] In brief, 126 mg Zirconium chloride (0.54 mM) was dissolved in 15 mL N,N-dimethylformamide and 1 mL concentrated hydrochloric acid was also added to the reaction bottle. After sufficient sonication, 147 mg 2,5-dihydroxyterephthalic acid (0.54 mM) was added to the mixed solution and continued sonication for 10 minutes. The dark brown solution reacts overnight at 80 °C and the yellow product was collected by centrifugation and washed three times with N,N-dimethylformamide, and anhydrous ethanol. The Uio-66(OH)$_2$ was activated at 80 °C before further experiments.

The synthesis of UiO-66@COP: 20 mg dried UiO-66(OH)$_2$ was added to a flask and dissolved with 5 mL acetonitrile and ultrasonic 10 min fully dispersed. Followed add 14 mg (0.04 mM) TAPB and 10 mg (0.04 mM) BCTA and ultrasonic 5 min. After thorough mixing, 0.5 mL of glacial acetic acid was added under magnetic stirring. Finally, the mixed solution was stirred at room temperature for 24 hours, and the yellow products were collected by centrifugation and washed with anhydrous ethanol and 1,4-dioxane three times.

The synthesis of UiO-66@HCOF: The washed UiO-66@COP was transferred to another flask and redispersed in 4 mL 1,4-dioxane and 1,2,3-trimethylbenzene mixed solution (v/v; 4/1).[2] After ultrasound dispersion, 0.6 mL deionized water, and 0.5 mL glacial acetic acid were added to the flask. Finally, the UiO-66@COP was aged at 70 °C for 48h without any disturbance. The yellow products were collected by centrifugation and washed with tetrahydrofuran three times.

Sample preparation and LDI-MS process: All the typical metabolite solutions and the substrate solution were prepared with deionized water. In detail, 1mg Val, Phen, Glc, Glu, Cre, His, Asp, and Arg dissolved in 1 mL deionized water and fully dissolved by ultrasound. In this work, in the preparation process, the sample was spotted on the target plate and after natural drying at room temperature, the substrate solution was deposited on the sample to form a homogeneous sample analyte. 1 μL typical metabolites solution was spotted on the target plate, and after drying at room temperature, 1 μL of the substrate solution was deposited on the dry sample spots. The serum samples were diluted ten times with deionized water before analysis and 800 nL of serum dilution was taken from each sample to spot on the target plate, and after drying at room temperature, 800 nL of substrate solution was deposited on the dry sample spots. all the LDI-MS analysis is completed on a MALDI-TOF MS (UlrafleXtreme MALDI-TOF/TOF MS) in a positive mode process and the laser energy is set at 79% and the analysis mode is the medium mode.

Statistical Analysis: The raw data acquired by UlrafleXtreme MALDI-TOF/TOF MS is exported from Flexanalysis 3.4 software. The pre-processing of the raw data including normalization, average, and alignment was achieved on the MALDI quant package on R.[3] Data were shown as the mean ± s.d. Univariate statistical analysis was completed on SPSS software version 20.0 (IBM Corp., Armonk, New York), including one-way anova for age comparison, and Chi-square test for sex comparison (Table S1). Significant differences were set at 0.05 (P<0.05). Orthogonal partial least squares discriminant analysis was performed on SIMCA 14.1 (MKS Umetrics, Umeå, Sweden). Random forest and the receiver operating characteristic (ROC) curves were generated at Metaboanalyst 4.0 (McGill University, Montreal, Canada, https:// www.metaboanalyst.ca/) and SPSS. The metabolite search was performed at Human Metabolome Database (https://hmdb.ca/).

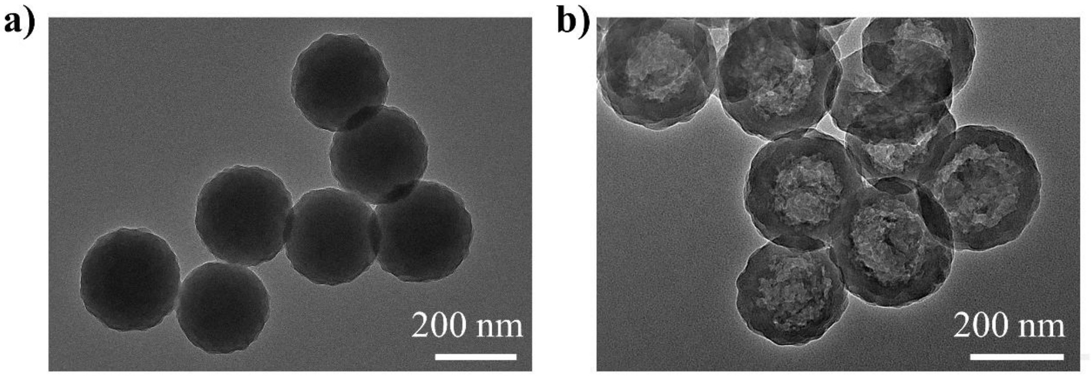

Figure S1. The TEM of COP, and HCOF aging for 48 hours.

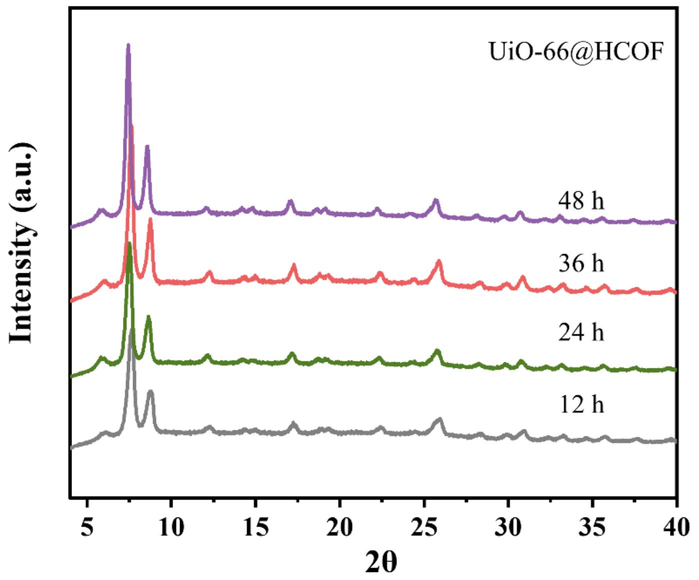

Figure S2. The XRD pattern of UiO-66@HCOF after aging for 12 h, 24h, 36h, and 48h.

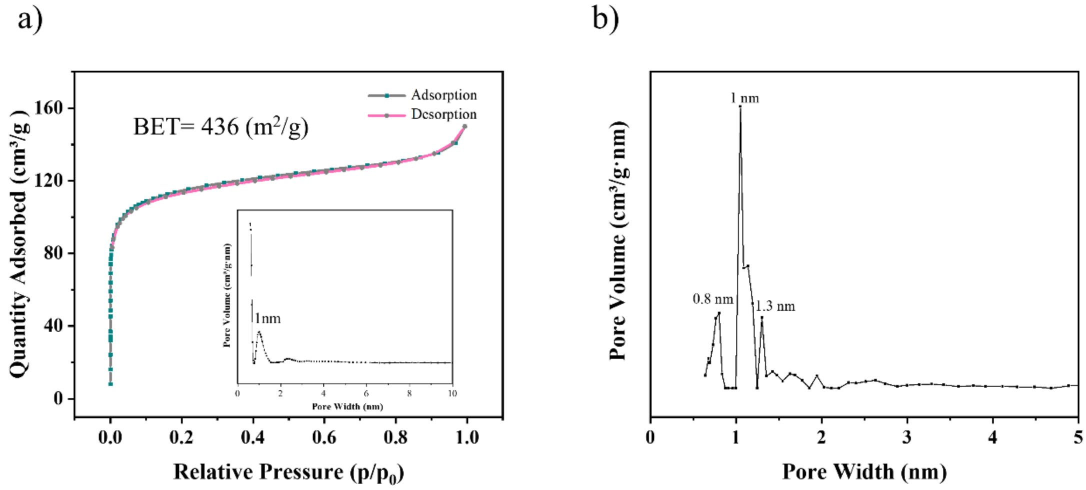

Figure S3. (a) The N_2 adsorption-desorption isotherm and the pore size distribution of UiO-66-$(OH)_2$. (b) The pore size distribution of UiO-66@HCOF.

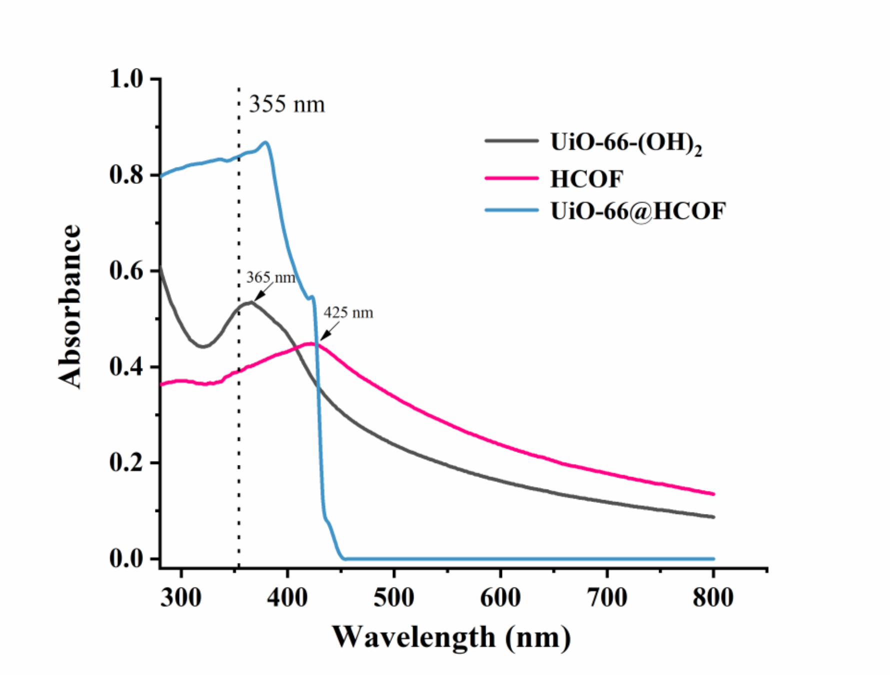

Figure S4. The UV-Vis spectra of UiO-66-$(OH)_2$, HCOF, and UiO-66@HCOF.

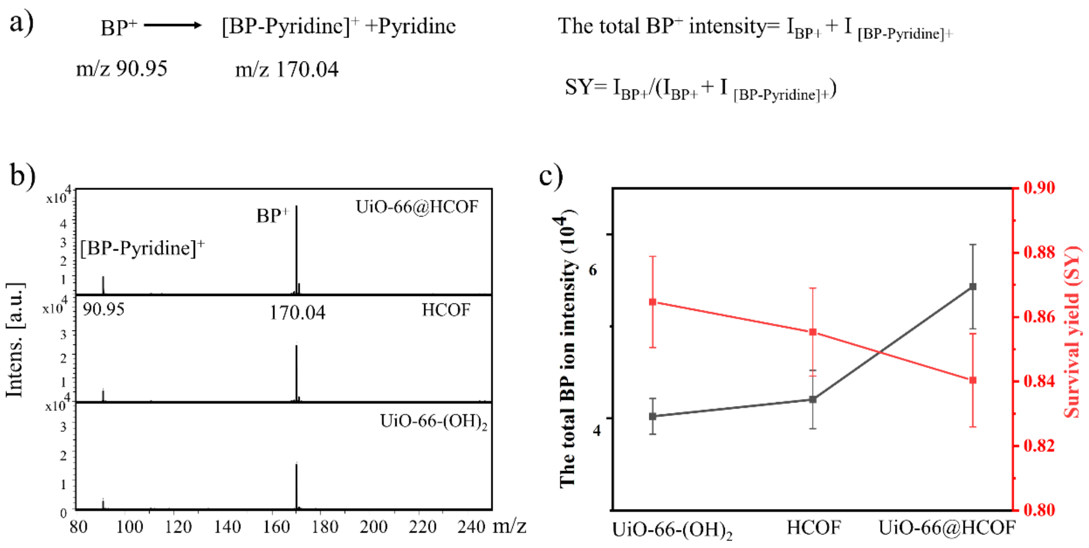

Figure S5. a) The decomposition of BP+ into [BP- Pyridine]$^+$ and Pyridine when the thermal transfer to the BP ions. b) The mass spectra of the analysis of BP$^+$ (2 mM) using UiO-66-$(OH)_2$, HCOF, and UiO-66@HCOF as substrate. c) The average of total BP ion intensity and the survival yield (n=10).

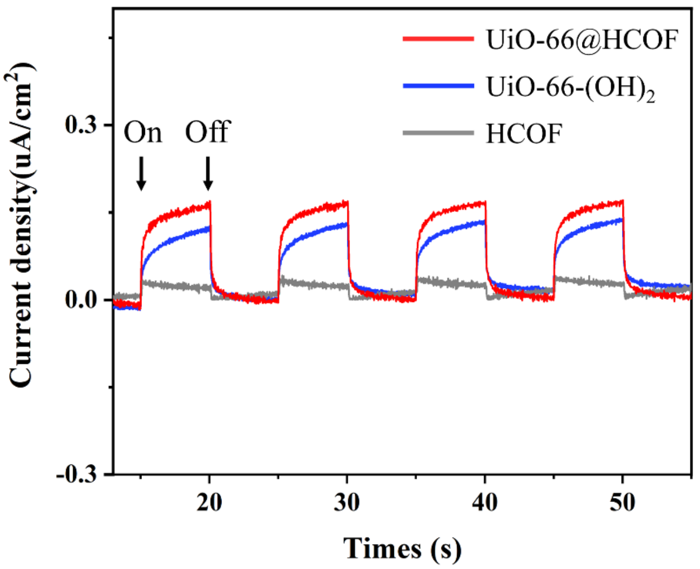

Figure S6. The photocurrent-time curves of UiO-66@HCOF, HCOF, and UiO-66-$(OH)_2$.

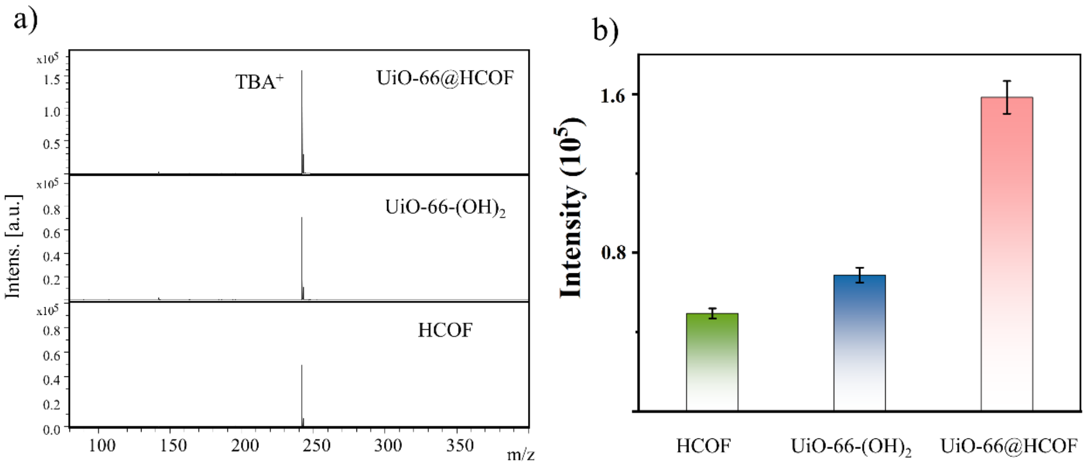

Figure S7. The mass spectra of TBA^+(a), and the average intensity of the TPB^+ using UiO-66@HCOF, HCOF, and UiO-66-$(OH)_2$ as substrate (n=10).

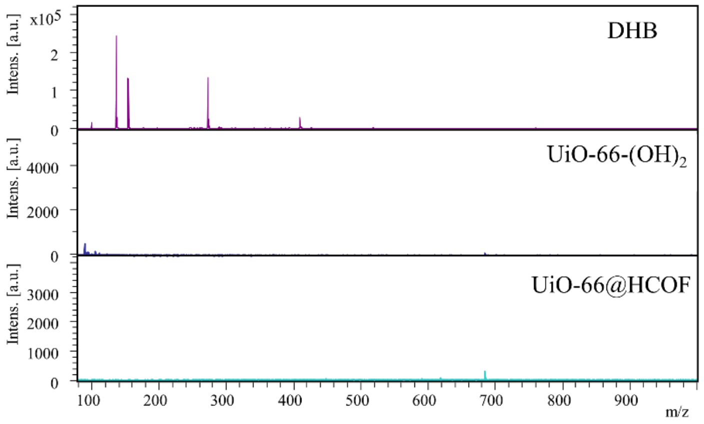

Figure S8. The mass spectra of the LDI-MS for the direct detection of single DHB (0.5 mg/mL), UiO-66-$(OH)_2$ (0.5 mg/mL), and UiO-66@HCOF (0.5 mg/mL).

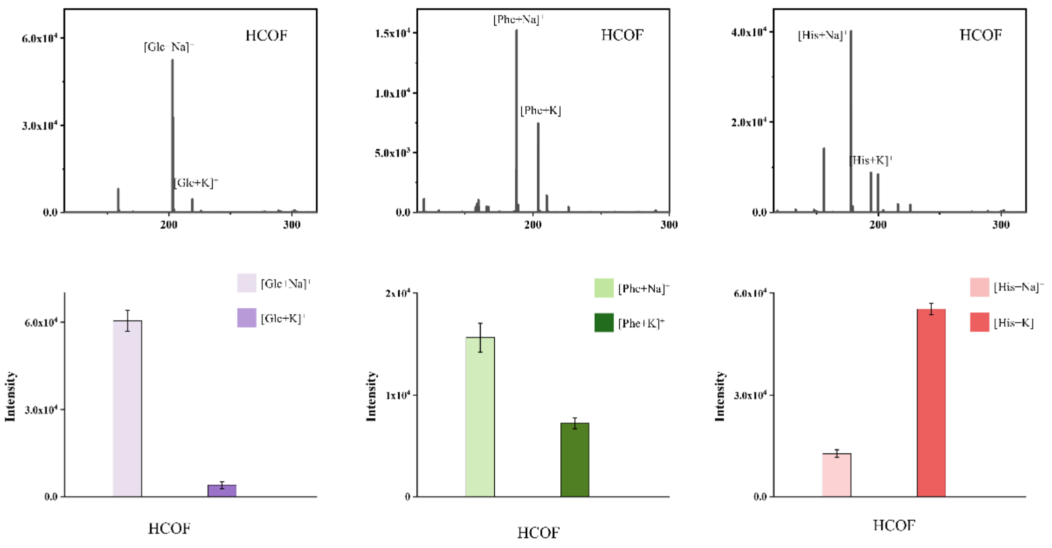

Figure S9. The detection of Glc(1 mg/mL), Phe(0.02mg/mL), and His(1 mg/mL) with HCOF as substrate and the corresponding Na^+/K^+ adducts.

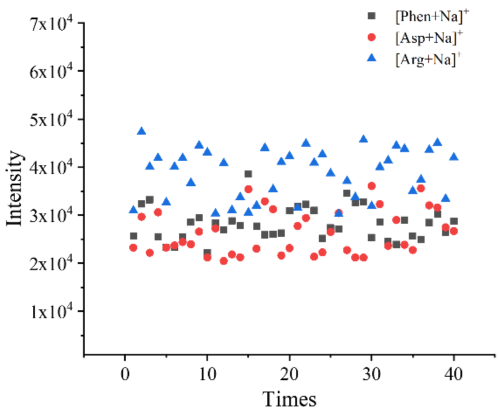

Figure S10. The LDI-MS with UiO-66@HCOF as substrate for the 40 times detection of the Phe (1 mg/mL), Asp (1 mg/mL), and Arg (1 mg/mL).

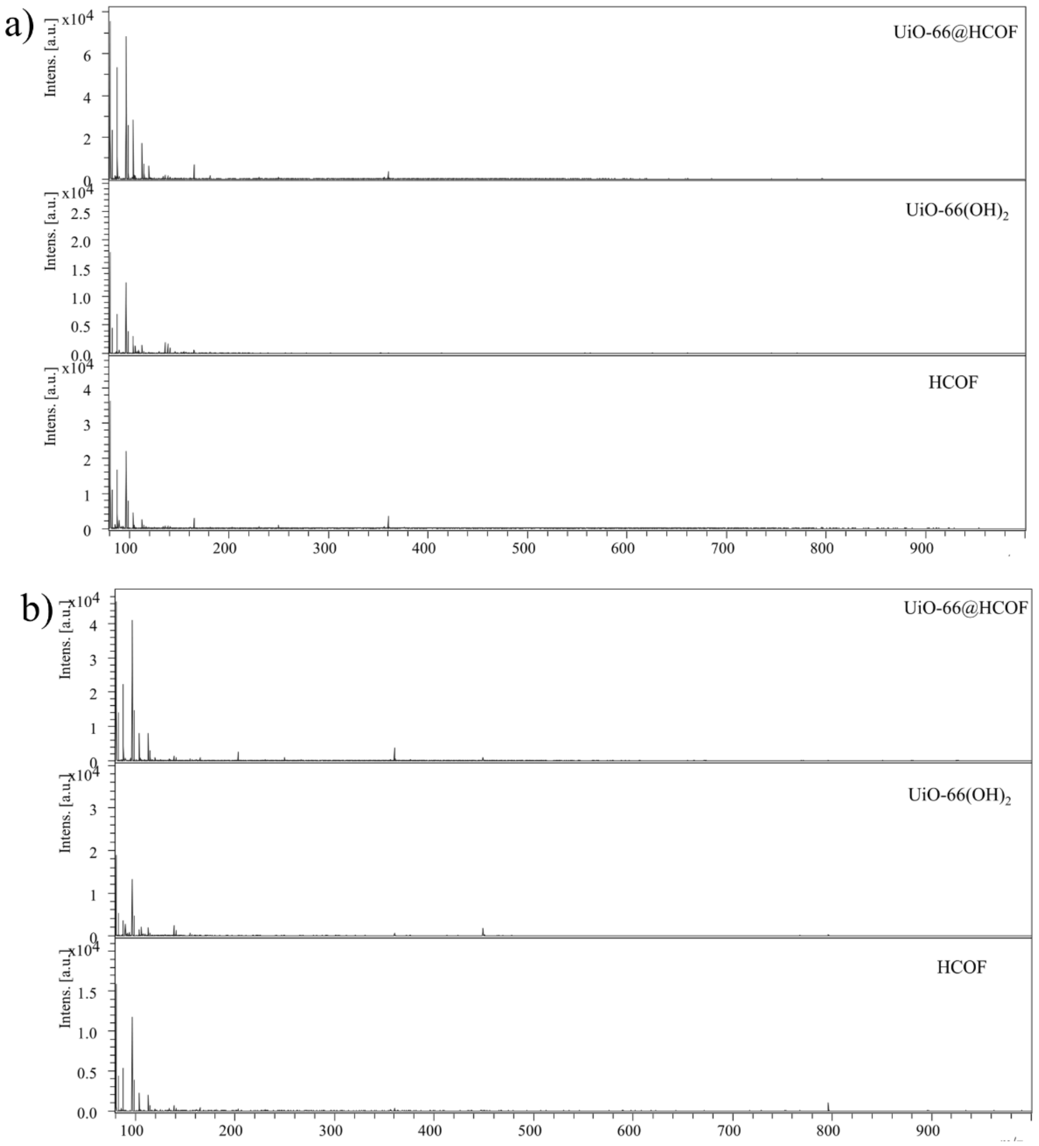

Figure S11. The typical serum metabolic profiling of a ACS patient (a) and a CON individual (b) by UiO-66@HCOF, UiO-66-$(OH)_2$, and HCOF.

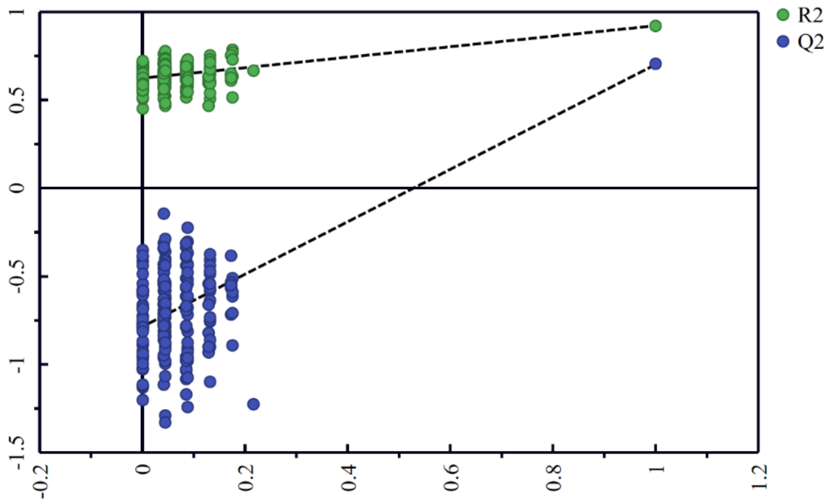

Figure S12. The 200 permutations of the OPLS-DA model between the NST, ST, UA, and CON.

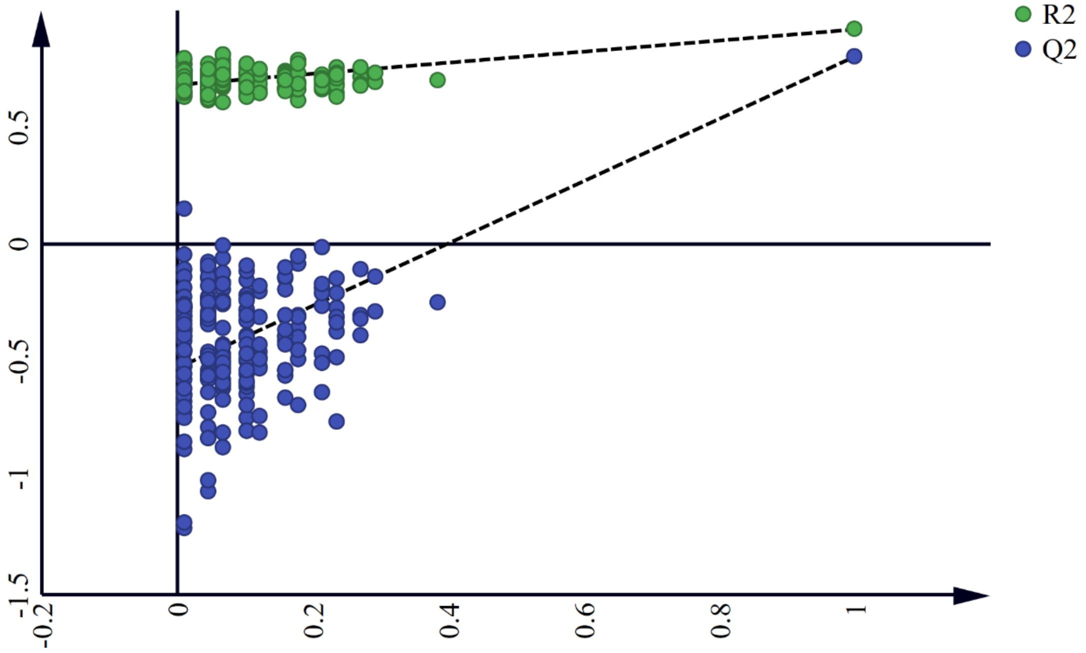

Figure S13. The 200 permutations of the OPLS-DA model between the NST and UA.

Table S1 Significant difference analysis of age/sex in AMI, UA, and CON

Characteristics		AMI	UA	*P* value	AMI	CON	*P* value	UA	CON	*P* value
Age(mean(±SD)		(59.83±12.244)	(60.64±8.593)	0.937[a]	(59.83±12.244)	(52.37±13.973)	0.002[a]	(60.64±8.593)	(52.37±13.973)	<0.001[a]
Sex	Male	80	35	<0.001[b]	80	27	<0.001[b]	35	27	0.917[b]
	Female	18	35		18	26		35	26	

[a)] *P* value was calculated by one-way anova; [b)] *P* value was calculated by Chi-square test.

Table S2. The list of the key features between UA and CON

Key signals (m/z)	Frequency(%)	*P* value
162.012	98	4.72E-12
221.002	96	1.03E-11
162.11	94	4.71E-08
176.042	82	5.36E-11
165.346	76	5.05E-13
166.959	70	1.87E-12
314.104	70	6.79E-05
147.926	66	3.83E-09

Table S3. The list of the key features between ACS and CON.

Key signals (m/z)	Frequency (%)	*P* value
619.322	97	1.98E-08
647.345	90	1.31E-09
381.2	90	1.18E-08
318.874	87	0.006637
165.346	73	6.33E-09
314.867	73	0.00711
353.172	73	7.57E-08
316.877	70	0.00592
263.082	60	0.044132

Table S4. The list of the key features between AMI and UA.

Key signals (m/z)	Frequency (%)	P value
180.911	96.7	2.20E-12
98.8582	83.3	6.10E-13
148.937	80.0	1.54E-09
360.268	76.7	8.75E-12
156.897	73.3	8.73E-09
154.881	70.0	9.99E-09
147.926	70.0	1.17E-11
196.918	70.0	6.45E-10
163.847	66.7	3.03E-08
96.8539	63.3	4.58E-13
122.947	60.0	2.28E-06
86.8948	60.0	1.04E-10

Table S5. The list of the key features between NST and ST.

Key signals (m/z)	Frequency（%）	P value
381.2	94	1.22E-16
685.232	88	1.45E-14
81.1466	86	7.18E-12
154.881	84	3.56E-15
142.926	84	6.75E-13
140.885	80	1.17E-16
138.883	78	3.84E-16
686.232	76	4.31E-13
109.968	76	1.23E-13
156.897	72	3.73E-14
123.992	70	5.59E-11

Table S6. The list of the key features between NST and UA.

Key signals (m/z)	Frequency（%）	P value
381.2	100	4.61E-16
620.324	92	2.60E-10
592.313	88	2.33E-10
619.322	88	1.81E-09
591.307	82	2.14E-09
647.345	82	8.90E-08
354.143	74	8.60E-07
687.229	62	3.95E-13
648.337	60	6.11E-08

Table S7. The list of the key features between AMI and CON.

Key signals (m/z)	Frequency（%）	P value
381.2	100	1.16E-10
647.345	100	6.53E-10
353.172	97	4.58E-08
318.874	97	0.000865
648.337	93	1.59E-09
316.877	90	0.000711
103.892	87	2.63E-09
199.004	83	2.90E-06
592.313	83	6.11E-08
619.322	80	1.08E-08
314.867	80	0.000916
620.324	77	4.14E-08
591.307	70	6.67E-08

Table S8. The list of the potential biomarkers.

Key signals	Assigned metabolite	Adduct	Compound ID	Delta (ppm)
162.11	L-Carnitine	M+H	HMDB0000062	15
147.926	Taurine	M+Na	HMDB0000251	526
156.897	Succinic acid	M+K	HMDB0000254	591
154.881	Fumaric acid	M+K	HMDB0000134	601
592.313	LysoPC	M+Na	HMDB0010403	41

[1] L. Chen, J. Ou, H. Wang, Z. Liu, M. Ye, H. Zou, *ACS Appl. Mater. Interfaces* **2016**, *8*, 20292.
[2] Z. Xiong, B. Sun, H. Zou, R. Wang, Q. Fang, Z. Zhang, S. Qiu, *J. Am. Chem. Soc.* **2022**, *144*, 6583.
[3] S. Gibb, K. Strimmer, *Bioinformatics* **2012**, *28*, 2270.
